# Electrode Based on the MWCNTs and Electropolymerized Thymolphthalein for the Voltammetric Determination of Total Isopropylmethylphenols in Spices

**DOI:** 10.3390/mi14030636

**Published:** 2023-03-11

**Authors:** Natalia Chernousova, Guzel Ziyatdinova

**Affiliations:** Analytical Chemistry Department, Kazan Federal University, Kremleyevskaya, 18, Kazan 420008, Russia

**Keywords:** chemically modified electrodes, voltammetry, electropolymerization, electropolymerization, phthalein dyes, thymol, carvacrol, spices, food analysis

## Abstract

Isopropylmethylphenols, namely thymol and carvacrol, are natural phenolic monoterpenoids with a wide spectrum of bioactivity making them applicable in the cosmetic, pharmaceutical, and food industry. The dose-dependent antioxidant properties of isopropylmethylphenols require their quantification in real samples. Glassy carbon electrode (GCE) modified with multi-walled carbon nanotubes (MWCNTs) and electropolymerized thymolphthalein has been developed for the sensitive quantification of isopropylmethylphenols. Conditions of thymolphthalein electropolymerization (monomer concentration, number of cycles, and electrolysis parameters) providing the best response to thymol have been found. Scanning electron microscopy and electrochemical methods confirm the effectivity of the electrode developed. The linear dynamic ranges of 0.050–25 and 25–100 µM for thymol and 0.10–10 and 10–100 µM for carvacrol with detection limits of 0.037 and 0.063 µM, respectively, have been achieved in differential pulse mode in Britton–Robinson buffer pH 2.0. The selectivity of the isopropylmethylphenols response in the presence of typical interferences (inorganic ions, saccharides, ascorbic acid) and other phenolics (caffeic, chlorogenic, gallic and rosmarinic acids, and quercetin) is a significant advantage over other electrochemical methods. The electrode has been used in the analysis of oregano and thyme spices. Total isopropylmethylphenols contents have been evaluated after a single sonication-assisted extraction with methanol.

## 1. Introduction

**Figure 1:**
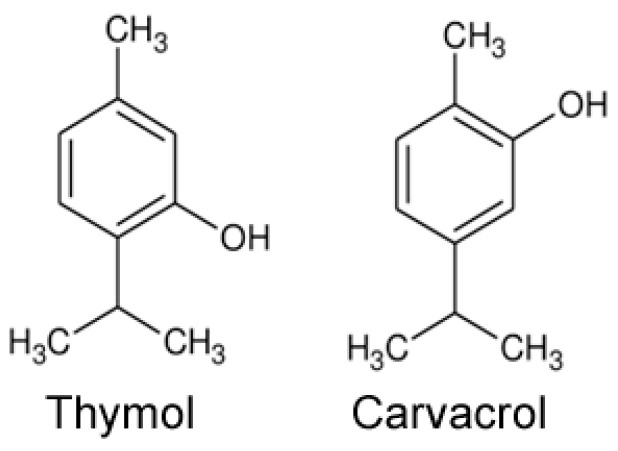
Structure of isopropylmethylphenols.

Isopropylmethylphenols, namely thymol and carvacrol (Figure 1), are phenolic monoterpenoids which are the major phenolic components of thyme, oregano, and other culinary and medicinal herbs [1].

Both thymol and carvacrol show a wide spectrum of biological activity such as antibacterial, antifungal, insecticidal, and antioxidant properties [2,3,4,5]. However, isopropylmethylphenols when presented in high concentrations demonstrate the pro-oxidant activity that is typical for natural phenolics [6]. Therefore, the quantification of isopropylmethylphenols is required to control of the real samples and their potential health effect.

The determination can be performed by UV-vis spectroscopy. Thymol is more investigated than carvacrol. Thymol determination is based on the diazotization-coupling reactions [7,8,9,10] or reactions with other reagents [11] with the following spectrophotometric detection. The determination of carvacrol in nanoemulsions is based on its absorption at 275 nm after recovery with acetonitrile [12]. Another spectrophotometric approach is based on the registration of the absorption of gold nanoparticles formed in the presence of carvacrol at pH 9.0 or both isopropylmethylphenols at pH 12.0 [13]. Insufficient selectivity and the necessity of derivatization are the major disadvantages of spectroscopic methods.

These limitations are overcome in chromatography which is usually used for the identification and quantification of isopropylmethylphenols. Thymol and carvacrol are volatile compounds. Therefore, gas chromatography with a flame ionization detector [14,15] or mass-spectrometric detection [16,17,18,19,20] is successfully applied for their determination in plant materials [14,20], biosamples [16], and foodstuffs [15,16,17,18,19]. High-performance liquid chromatography with UV- [21,22,23,24,25,26] and fluorimetric detection [27] has been widely applied in the analysis of liquid samples such as essential oils, medicinal plant extracts, and honey. Recently, ultraperformance convergence chromatography with UV detection has been developed for the determination of thymol and carvacrol in *Thymi herba* [28]. Such an approach significantly decreases the use of organic solvents and improves the separation time (less than 2.5 min) and resolution of the peaks. Another original greener approach to thymol quantification is based on the application of normal-phase high-performance thin-layer chromatography with a binary combination of cyclohexane and ethyl acetate (85:15, *v*/*v*) as a mobile phase and anisaldehyde-sulfuric acid as a derivatizing/visualizing agent [29].

The presence of a phenolic moiety in the thymol and carvacrol structure makes them electroactive. Therefore, high-performance liquid chromatography with electrochemical detection has been shown to be an effective tool for the determination of isopropylmethylphenols in phytopharmaceuticals, culinary herbs, and foodstuffs [30,31,32].

In spite of high selectivity of chromatographic methods, the preliminary concentration and liquid or solid phase extraction of isopropylmethylphenols from real samples is usually required [15,16,17,19,22,23,24,25,26]. These steps complicate the analysis and require the application of additional reagents, solvents, and equipment, making the determination procedure tedious and more expensive. Furthermore, fast screening of the samples outside the laboratory cannot be realized.

Electrochemical methods, in particular voltammetry, are a good alternative to chromatography. The possibility of miniaturization, direct analysis, cost-efficiency, and rapidity in combination with high accuracy and sufficient selectivity are the most attractive advantages over other instrumental methods. Various voltammetric approaches have been developed for the isopropylmethylphenols quantification. The similarity of the thymol and carvacrol structure leads to almost similar oxidation potentials of both compounds. Therefore, total isopropylphenols determination is usually performed. Individual quantification is possible for the sample containing only one of the isopropylmethylphenols or with the application of chemometric treatment of voltammetric data for the mixtures [33,34,35].

Traditional carbon-based electrodes [36,37,38,39], boron-doped diamond electrodes [40,41], and platinum microelectrode [42] have been applied for the quantification of total isopropylphenols. However, the sensitivity of the response can be further improved. Various types of electrode surface modifiers have been developed for the improvement of the isopropylphenols determination sensitivity. Carbon nanomaterials (multi- [43,44,45] and single-walled carbon nanotubes [46], graphene oxide nanosheets [47]), metal oxide nanoparticles (CeO_2_ nanoparticles in combination with graphene [48] and Brij^®^ 35 micellar medium [49], La_2_O_3_/Co_3_O_4_ nanocomposite [50]), monodisperse Ag@C@Ag core-double shell spheres [51], and MnY nanozeolite [52] have been successfully used as sensing layers of electrodes for thymol and carvacrol. Thymol is more often studied compared to carvacrol.

Among a wide range of electrode surface modifiers, polymeric coverages received attention in the electroanalysis of natural phenolics [53,54]. However, polymer-modified electrodes are almost completely out of consideration in application to isopropylmethylphenols. The detection of carvacrol in essential oils has been performed using its electropolymerization at the copper electrode [55]. Unfortunately, quantification of carvacrol is impossible. The only example reported to date is the polyacrylamide-embedded graphite-molecular-imprinted polymer-based electrode for thymol determination [56]. The analytical characteristics achieved are among the best ones reported to date for the voltammetric determination.

Further development in the field of polymer-modified electrodes for the isopropylmethylphenols can be focused on the application of polymeric coverages based on the electropolymerized dyes. Recently, electrodes based on the electropolymerized azo [57] and triphenylmethane dyes [58,59,60,61] have been developed for the determination of phenolic acids and flavonoids. Triphenylmethane dyes containing phenolic fragments form non-conductive polymers. Therefore, the electropolymerization of these dyes has been carried out at the surface of the electrodes modified with carbon nanomaterials [58,59,60,61], which provide sufficient conductivity of the electrode and high surface area available for the electrodeposition of polymer.

The current work is focused on the development of sensitive voltammetric method for the isopropylmethylphenols determination using electrode modified with multi-walled carbon nanotubes (MWCNTs) and electropolymerized thymolphthalein. The conditions of electropolymerization have been optimized on the basis of thymol response. The electrooxidation parameters of thymol and carvacrol at the modified electrode have been calculated. The electrode developed has been successfully tested and validated on the extracts of oregano and thyme spices.

## 2. Materials and Methods

### 2.1. Reagents

Thymol (99.5% purity) and thymolphthalein (95%) from Sigma (Steinheim, Germany) and carvacrol (98%) from Aldrich (Steinheim, Germany) were used. Their standard 10 mM solutions were prepared in methanol (c.p. grade) in 5.0 mL flasks. Ascorbic (99%), gallic (99%), caffeic (98%) and rosmarinic (98%) acids, quercetin dihydrate (95%) from Sigma (Steinheim, Germany), and chlorogenic acid (95%) from Aldrich (Steinheim, Germany) have been used in the interference study. Their 10 mM stock solutions in methanol were prepared in 5.0 mL flasks. The exact dilution was used for the preparation of less concentrated solutions.

MWCNTs (outer diameter 40–60 nm, inner diameter 5–10 nm and 0.5–500 μm length) from Aldrich (Steinheim, Germany) were used as a platform for further electrodeposition of polythymolphthalein. A homogeneous 0.5 mg mL^−1^ suspension of MWCNTs was prepared in 1% aqueous solution of sodium dodecyl sulfate (Panreac, Barcelona, Spain) by 30 min of sonication in an ultrasonic bath (WiseClean WUC-A03H (DAIHAN Scientific Co., Ltd., Wonju-si, Republic of Korea)).

All other reagents were c.p. grade and used as received.

The laboratory temperature was (25 ± 2 °C).

### 2.2. Equipment

Electrochemical measurements were conducted on the potentiostat/galvanostat Autolab PGSTAT 302N with the FRA 32M module (Eco Chemie B.V., Utrecht, The Netherlands) and NOVA 1.10.1.9 software (Eco Chemie B.V., Utrecht, The Netherlands). A glassy electrochemical cell of 10 mL volume was used. The tree-electrode system consisted of a working glassy carbon electrode (GCE) of 3 mm diameter (CH Instruments, Inc., Bee Cave, TX, USA), or a modified electrode, an Ag/AgCl (3 M KCl) reference electrode, and a platinum wire as an auxiliary electrode.

The pH measurements were carried out using the “Expert-001” pH meter (Econix-Expert Ltd., Moscow, Russia) with a glassy electrode.

Spectrophotometric measurements were performed on the spectrophotometer PE-5300 (NPO Ecros, Saint Petersburg, Russia).

A high-resolution field emission scanning electron microscope Merlin^TM^ (Carl Zeiss, Oberkochen, Germany), operated at an accelerating voltage of 5 kV and an emission current of 300 pA, was used for the electrode surface morphology characterization.

### 2.3. Electrode Surface Modification

The GCE surface was mechanically renewed after each measurement. The electrode was polished on the 0.05 µm alumina slurry and rinsed with acetone and double-distilled water. Then, 2 μL of 0.5 mg mL^−1^ MWCNTs suspension was placed at the GCE surface and kept for 7 min at room temperature for the evaporation of the solvent to dryness.

Potentiodynamic electrolysis was applied for the electrodeposition of polythymolphthalein layer formation. Five scans of the supporting electrolyte (phosphate buffer pH 7.0) were recorded to achieve a stable blank curve. Then, the electropolymerization of thymolphthalein was performed. The supporting electrolyte pH, concentration of the monomer, number of scans, polarization window and potential scan rate were optimized using oxidation currents of thymol as a control parameter.

Polymer-modified electrode was thoroughly rinsed with distilled water and used for further measurements.

### 2.4. Electrochemical Measurements

Voltammetric measurements were carried out in Britton–Robinson buffer (BRB) in the pH range of 2.0–12.0. Five scans were recorded prior to the analytes addition. Then, an aliquot portion of thymol or a carvacrol solution was inserted in the electrochemical cell, and cyclic voltammograms (CVs) were recorded from 0.0 to 1.0 V or from 0.0 to 1.3 V at the potential scan rate of 100 mV s^−1^. Differential pulse voltammograms (DPVs) were recorded from 0.0 to 1.2 V at a potential scan rate of 20 mV s^−1^. The modulation parameters were preliminary optimized as modulation amplitude of 100 mV and modulation time of 25 ms. Baseline correction using the NOVA 1.10.1.9 software (Eco Chemie B.V., Utrecht, The Netherlands) was used.

Chronoamperometric curves were registered at 0.45 V for 75 s using 1.0 and 2.0 mM ferrocyanide ions in 0.1 M KCl.

Electrochemical impedance spectroscopy (EIS) was performed in the presence of a redox probe (1.0 mM ferri-/ferrocyanide ions) in 0.1 M KCl in the frequency range of 10 kHz to 0.04 Hz (12 points per decade) with the amplitude of sine potential of 5 mV. The polarization potential of 0.23 V was used. It was calculated as a half sum of redox probe peak potentials on the CVs recorded at the polythymolphtalein-modified electrode. Randles’ equivalent circuit was used for the EIS spectra fitting using NOVA 1.10.1.9 software (Eco Chemie B.V., Utrecht, The Netherlands).

### 2.5. Real Samples Analysis

Commercially available oregano and thyme spices were used. Sonication-assisted extraction of isopropylmethylphenols with methanol was used. Briefly, the exact weight of the sample (0.1000 ± 0.0002 g) was put in the 5 mL sealed tube, and a certain volume of the extragent (2.0–5.0 mL) was added. The tube was placed to the sonication bath. Spice: extragent ratio and extraction time were varied to find the best recovery of isopropylmethylphenols. The extracts obtained were filtered through the paper filter, filled with methanol to the initial volume (2.0–5.0 mL), and used for further measurements.

Voltammetric determination of isopropylmethylphenols was performed in BRB pH 2.0. Aliquot of the extract (50 µL) was added to the electrochemical cell (total volume of solution was 5.0 mL) and DPVs were recorded from 0.0 to 1.2 V at the modulation amplitude of 100 mV and the modulation time of 25 ms with the potential scan rate of 20 mV s^−1^. Total isopropylmethylphenol contents was calculated using calibration graph for thymol, giving more sensitive response compared to carvacrol.

Spectrophotometric measurements of total isopropylmethylphenols were performed according to a known procedure [13].

### 2.6. Statistical Analysis of the Data

Five replicates (three replicates for spectrophotometry) were performed. The statistical treatment of data was completed with a confidence level of 0.95. All results were expressed as the average value with the confidence interval. The relative standard deviation was used for the characterization of random errors of determination. The *F*- and *t*-tests were applied to validate the method developed.

The detection limits were calculated as 3SD_a_/*b*, where SD_a_ was the standard deviation of the calibration graph intercept and *b*—the calibration graph slope.

Regression analysis was performed using the OriginPro 8.1 software (OriginLab, Northampton, MA, USA).

## 3. Results and Discussion

### 3.1. Potentiodynamic Electropolymerization of Thymolphthalein at the MWCNTs-Modified Electrode

Thymolphthalein is an electrochemically active dye due to the presence of thymol fragments. Potentiodynamic electropolymerization of thymolphthalein has been performed in neutral medium using phosphate buffer as the supporting electrolyte. The choice of pH is based on the dye properties at various pH values. Thymol moieties are easily oxidized by air oxygen in the basic medium while protonation of thymolphthalein molecule takes place in acidic medium that makes electron detachment more complicated.

**Figure 2:**
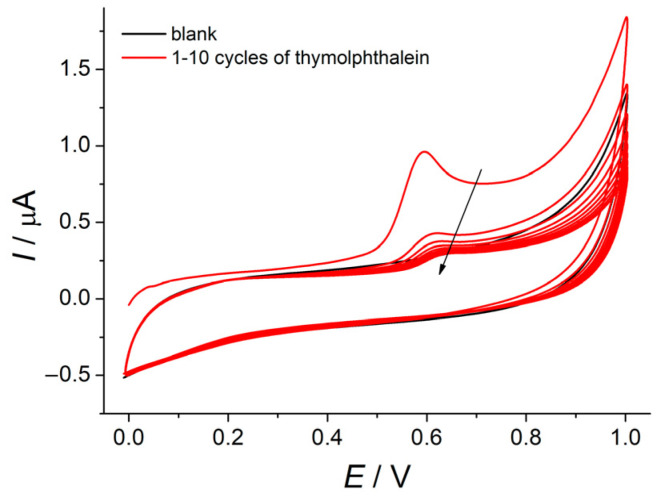
CVs of 10 µM thymolphthalein at the MWNTs/GCE in phosphate buffer pH 7.0 (scans 1–10). The potential scan rate is 100 mV s^−1^. The arrow shows the changes of the thymolphthalein oxidation currents with increase of the number of cycles.

Thymolphthalein is irreversibly oxidized at 0.594 V at MWCNTs/GCE in phosphate buffer pH 7.0 (Figure 2).

**Scheme 1:**
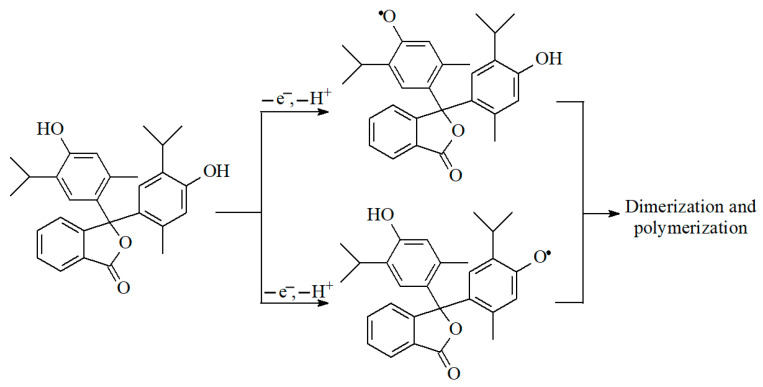
Electrooxidation of thymolphthalein.

One electron and one proton are involved in the reaction with the formation of phenoxyl radicals (Scheme 1), which is in line with the reported for thymolphthalein [59] and bromothymol blue [62]. The radicals formed can undergo the following dimerization and polymerization reactions (Scheme 1). This process is confirmed by the CVs on the second and subsequent scans at which a significant decrease in the dye oxidation currents has been observed. Thus, thymolphthalein electropolymerization proceeds with the formation of non-conductive coverage that is typical behavior for the phenolic monomers [54,63] including triphenylmethane dyes such as aluminon [58], pyrocatechol violet [60], bromocresol purple [61].

MWCNTs amount used as a substrate for the polymer electrodeposition affects thymolphthalein response. The volume of drop-casted MWCNTs suspension cannot be changed due to the incomplete coverage or spread beyond the working surface of the electrode for smaller and larger volumes, respectively. Increase the concentration of MWCNTs in suspension makes it less stable. Furthermore, a partial leaching of the modifier from the electrode surface occurs after its immersion in the supporting electrolyte solution.

**Figure 3:**
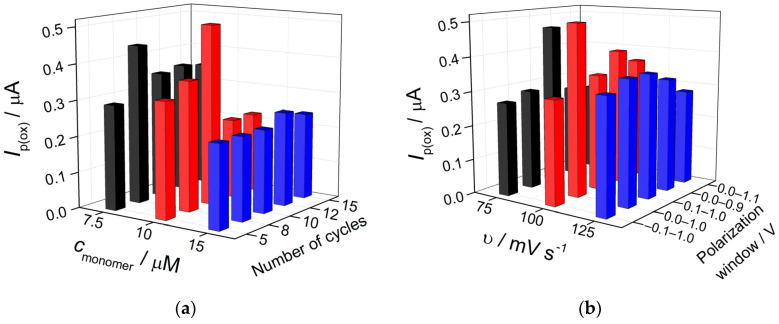
Effect of thymolphthalein electropolymerization conditions on the oxidation currents of 10 µM thymol at the polymer-modified electrode using cyclic voltammetry: (**a**) effect of the monomer concentration and number of scans; (**b**) effect of the electrolysis parameters (polarization window and potential scan rate).

The electropolymerization conditions for obtaining a polythymolphthalein layer have been optimized using a response of 10 µM thymol at the polymer-modified electrode. Oxidation potential changes insignificantly, whereas peak currents are statistically significantly changed. Therefore, the last ones have been considered as a control parameter (Figure 3).

Thymol oxidation currents are strongly affected by monomer concentration and a number of scans used for the polymeric coverage obtaining (Figure 3a). A 15 µM monomer concentration leads to significantly lower thymol oxidation currents than for the 7.5 and 10 µM concentration. Moreover, the change in the number of scans almost does not result in the difference in the thymol oxidation currents. This is probably caused by a higher thickness of the non-conducting polymer which partially blocks the electron transfer. The highest oxidation currents of thymol have been observed at the polymer obtained from 10 µM monomer at 10 scans. A further increase in the scans number leads to the formation of thicker coverage and electron transfer blockage.

The effect of electrolysis parameters used for the polymerization of monomer on the oxidation currents of thymol has been studied (Figure 3b). The highest response of thymol has been observed at the polymeric coverage obtained by potential cycling from 0.0 to 1.0 V with the scan rate of 100 mV s^−1^. Expanding or narrowing the polarization window leads to the statistically significant decrease in thymol oxidation currents that is probably caused by the electrolysis time.

Thus, the optimal conditions of thymolphthalein electropolymerization are 10 µM monomer and 10 potential scans from 0.0 to 1.0 V at a scan rate of 100 mV s^−1^ in phosphate buffer pH 7.0.

### 3.2. Surface and Electron Transfer Characterization of the Electrodes

#### 3.2.1. Electrodes Surface Morphology

**Figure 4:**
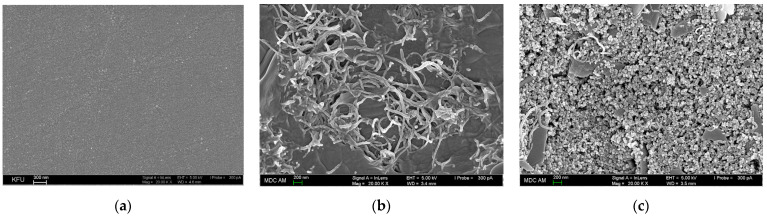
Surface morphology of the electrodes based on the scanning electron microscopy data: (**a**) Bare GCE; (**b**) MWCNTs/GCE; (**c**) Polythymolphthalein/MWCNTs/GCE.

The surface of the bare GCE and modified electrodes has been studied by scanning electron microscopy (Figure 4). The presence of MWCNTs (Figure 4b) and polymeric coverage (Figure 4c) significantly changes electrode surface morphology compared to bare GCE. MWCNTs are strongly intertwined and included in the film of sodium dodecyl sulfate used as a dispersive agent for MWCNTs (Figure 4b). The polythymolphthalein layer forms a porous coverage from spheroid particles of 23–50 nm diameter that are evenly distributed at the electrode surface (Figure 4c). These data agree with [59]. Polymeric coverage provides high roughness and area of the electrode surface.

#### 3.2.2. Evaluation of the Electroactive Surface Area and Electron Transfer Properties of Electrodes

**Figure 5:**
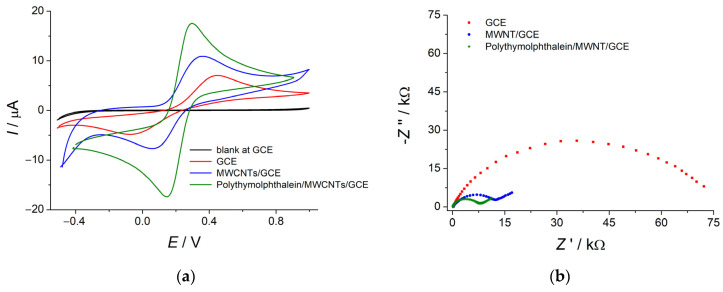
(**a**) CVs of 1.0 mM ferrocyanide ions in 0.1 M KCl at the bare GCE, MWCNTs/GCE and polythymolphthalein/MWCNTs/GCE (υ = 100 mV s^−1^); (**b**) nyquist plot for bare GCE, MWCNTs/GCE and polythymolphthalein/MWCNTs/GCE in the presence of 1.0 mM ferri-/ferrocyanide ions in 0.1 mol L^−1^ KCl. *E* = 0.23 V; frequency range is 10 kHz–0.04 Hz; amplitude is 5 mV.

Electrochemical evaluation of the electroactive surface area of the electrodes has been performed using ferrocyanide ions as a standard redox probe (Figure 5a). Electrooxidation of ferrocyanide ions at the bare GCE shows a lack of reversibility. Electrode surface modification provides a significant increase in the electrochemical system reversibility as the peak potential separation indicates. Ferrocyanide ions oxidation currents are also increased vs. those at the bare GCE. These data confirm a higher electron transfer rate at the modified electrode that is also confirmed by the EIS data (Figure 5b).

Electroactive surface area of the bare GCE has been calculated using chronoamperometric data at 0.45 V (Figure S1) and Cottrell equation [64]. Cyclic voltammetry results and the Randles–Ševčík equation [64] have been used for the modified electrodes. The electroactive surface area of the electrodes is presented in Table 1.

Electron transfer characterization has been performed by EIS in the presence of a 1.0 mM mixture of ferri-/ferrocyanide ions as a redox probe. The corresponding Nyquist plots are shown in Figure 5b. The modified electrodes exhibit improvement in the electron transfer compared to bare GCE as confirmed by a significantly lower semicircle diameter at high frequencies. The polythymolphthalein-modified electrode is characterized by the lowest electron transfer resistance among the electrodes under consideration that agree with cyclic voltammetry data. Similar behavior has been reported for the poly(bromocresol purple)-modified electrode [61]. The impedance spectra fitting has been carried out using Randles’ equivalent circuits (Table 1). A χ^2^ value confirms a high accuracy of the spectra fitting.

A 10-fold lower electron transfer resistance for polythymolphthalein-modified electrode vs. bare GCE (1.66-fold decrease vs. MWCNTs/GCE) clearly confirms effectivity of polymeric coverage in the electron transfer and applicability in electroanalysis.

### 3.3. Voltammetric Characteristics of Thymol at the Bare and Modified GCE

**Figure 6:**
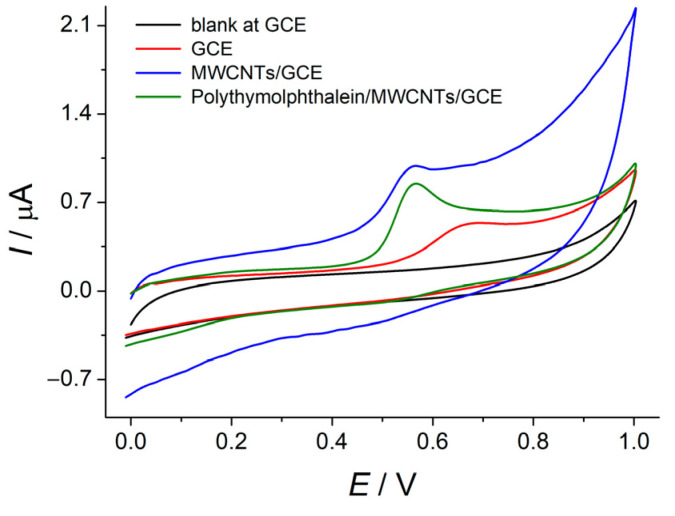
CVs of 10 µM thymol at the bare GCE, MWCNTs/GCE, and polythymolphthalein/MWCNTs/GCE in Britton–Robinson buffer pH 7.0. The potential scan rate is 100 mV s^−1^.

The voltammetric behaviour of isopropylmethylphenols at the bare and modified electrodes has been studied with the example of thymol in Britton-Robinson buffer pH 7.0. An irreversible oxidation peak at 0.655 V has been recorded at the bare GCE (Figure 6). Oxidation currents are low enough and significantly drop down as the thymol concentration is decreased.

Changes in the voltammetric characteristics of thymol have been observed at the MWCNTS/GCE. Oxidation potential is shifted on 91 mV to the less positive value, which is caused by the electrocatalytic effect of MWCNTs. Similar behavior has been reported for GCE modified with carboxylated MWCNTs dispersed in sodium dodecyl sulfate [44]. Thymol oxidation currents are 1.6-fold higher than at the bare GCE due to the increase in the electroactive surface area of the modified electrode. However, the MWCNTs-modified electrode shows high capacitive currents. Polymer-modified electrode demonstrates much lower capacitive currents and the well-shaped response of thymol. The oxidation potential is the same as at MWCNTS/GCE, but the oxidation currents are 1.9-fold higher (0.50 ± 0.02 and 0.26 ± 0.01 µA for polythymolphthalein/MWCNTs/GCE and MWCNTs/GCE, respectively). This effect is explained by an increase in the electroactive surface area of the polymer-modified electrode. Thus, polythymolphthalein/MWCNTs/GCE provide sensitive response to thymol and can be used for the determination of isopropylmethylphenols.

### 3.4. Electrooxidation of Isopropylmethylphenols at the Polythymolphthalein-Modified Electrode

**Figure 7:**
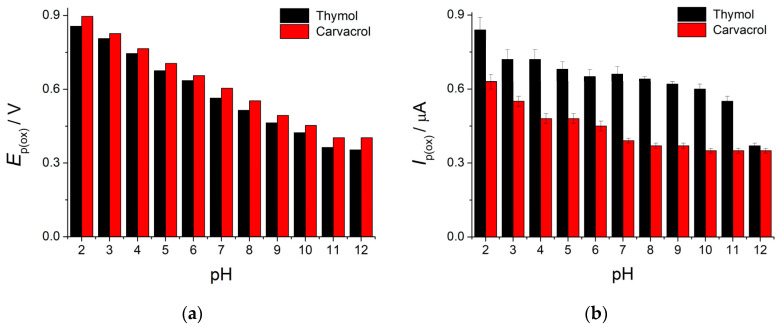
Effect of Britton–Robinson buffer pH on the voltammetric characteristics of 25 µM isopropylmethylphenols at the polythymolphthalein/MWCNTs/GCE: (**a**) changes in the oxidation potentials; (**b**) changes in the oxidation currents.

Electrooxidation of thymol and carvacrol has been studied by cyclic voltammetry. The effect of Britton–Robinson buffer in the pH range from 2.0 to 12.0 on the voltammetric characteristics of isopropylmethylphenols has been evaluated (Figure 7).

Well-defined oxidation peaks of both thymol and carvacrol have been registered in the whole pH range. The oxidation potentials are gradually shifted to lower values as the pH is increased up to pH 11.0 (Figure 7a). Such behavior confirms the participation of protons in electrode reactions. At pH > 11, the oxidation potentials become independent of pH and agree well with the ionization constants of isopropylmethylphenols (pKa are 10.59 for thymol and 10.38 for carvacrol [65]).

The oxidation potentials of thymol and carvacrol are linearly dependent on the pH in the range of 2.0 to 11.0 (Equations (1) and (2) for thymol and carvacrol, respectively).

*E* [V] = (0.962 ± 0.007) − (0.055 ± 0.001)pH  *R*^2^ = 0.9970, (1)

*E* [V] = (0.987 ± 0.008) − (0.054 ± 0.001)pH  *R*^2^ = 0.9965, (2)

The slopes are close to a theoretical value of 0.059, allowing the conclusion that an equal number of protons and electrons participate in the electrooxidation of isopropylmethylphenols.

The oxidation currents of thymol and carvacrol decreased as pH increased. Moreover, carvacrol oxidation currents are statistically significantly lower than that for thymol. The highest oxidation currents of both isopropylmethylphenols have been obtained at a pH 2.0. This effect is explained by the stability of thymol and carvacrol in an acidic medium so far as oxidation by air oxygen does not take place. Therefore, further investigations have been carried out at pH 2.0.

**Figure 8:**
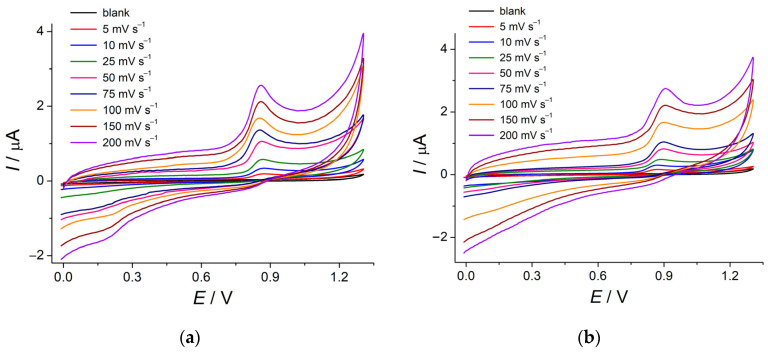
CVs of 25 µM isopropylmethylphenols at the polythymolphthalein/MWCNTs/GCE in Britton-Robinson buffer pH 2.0 at various potential scan rates: (**a**) thymol; (**b**) carvacrol.

The effect of the potential scan rate on the voltammetric characteristics of isopropylmethylphenols has been evaluated (Figure 8) in order to know the electrooxidation parameters.

Electrooxidation of thymol and carvacrol is a diffusion-controlled process, as confirmed by the linear dependence of oxidation currents vs. the square root of the potential scan rate and the slope of ln*I* = *f*(lnυ) close to 0.50 [64] (Equations (3) and (4) for thymol and Equations (5) and (6) for carvacrol).

*I* [µA] = (−0.14 ± 0.02) + (0.096 ± 0.002) υ^½^ [mV s^−1^]  *R*^2^ = 0.9957, (3)

ln*I* [µA] = (1.28 ± 0.04) + (0.65 ± 0.01) lnυ [V s^−1^]  *R*^2^ = 0.9971, (4)

*I* [µA] = (−0.069 ± 0.007) + (0.0685 ± 0.0008) υ^½^ [mV s^−1^]  *R*^2^ = 0.9990, (5)

ln*I* [µA] = (0.92 ± 0.03) + (0.615 ± 0.008) lnυ [V s^−1^]  *R*^2^ = 0.9988. (6)

Both isopropylmethylphenols are oxidized irreversibly, which is confirmed by the absence of cathodic peaks on the cyclic voltammograms. Similar behavior has been reported at other electrodes including those that are chemically modified [40,41,44,45,48,49,50,51,52,56]. The anodic transfer coefficients of 0.40 and 0.44 for thymol and carvacrol, respectively, have been calculated from the slopes of the Tafel plots at low potential scan rates [64]. The number of electrons participating in the electrooxidation of isopropylmethylphenols has been calculated using Equation (7) [64] as follows:

∆*E*_1/2_ = 47.7/α_a_*n*, (7)

where ∆*E*_1/2_ is the difference between the oxidation potential and the half-wave potential, α_a_ is the anodic transfer coefficient, and *n* is the number of electrons. Thus, 2.2 ± 0.1 and 2.3 ± 0.1 electrons are involved in the electrode reaction of thymol and carvacrol, respectively.

**Scheme 2:**
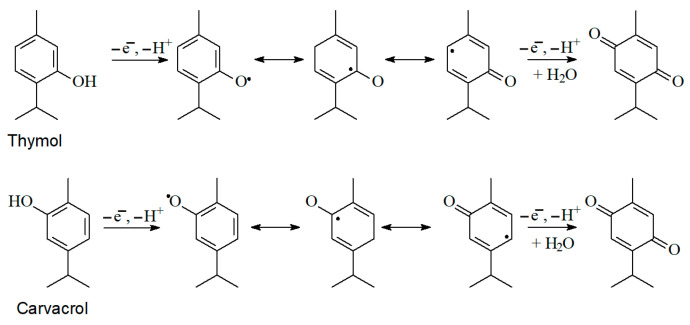
Electrooxidation of isopropylmethylphenols.

Summarizing the data obtained, two electrons and two protons participate in the electrooxidation of isopropylmethylphenols that agree well with values reported for thymol at CeO_2_ nanoparticles modified GCE in Brij^®^ 35 micellar medium [49]. The oxidation of thymol and carvacrol to the thymoquinone occurs at the electrode surface (Scheme 2), which is similar to reactions of structurally related phenols [66,67].

### 3.5. Differential Pulse Voltammetric Determination of Isopropylmethylphenols

**Figure 9:**
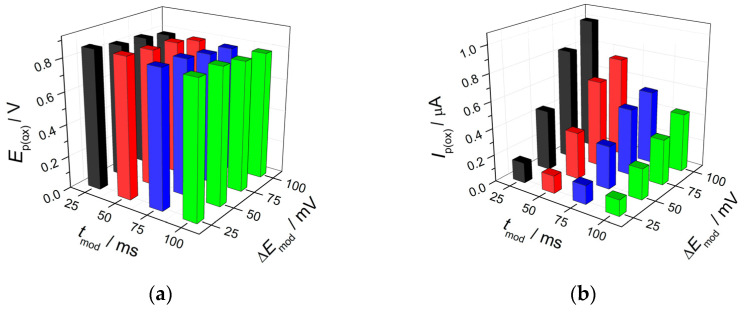
Effect of the modulation parameters on the voltammetric characteristics of 10 µM thymol at the polythymolphthalein/MWCNTs/GCE in Britton-Robinson buffer pH 2.0: (**a**) oxidation potential; (**b**) oxidation current. The potential scan rate is 20 mV s^−1^.

A determination of thymol and carvacrol has been performed using differential pulse voltammetry which provides a higher sensitivity to the target analytes response compared to linear sweep mode. The effect of the modulation parameters on the response of isopropylmethylphenols at the polythymolphthalein/MWCNTs/GCE in the Britton–Robinson buffer pH 2.0 has been studied using thymol as a standard (Figure 9).

The oxidation potential of thymol gradually decreased with increasing modulation amplitude and time (Figure 9a). The oxidation currents become higher as the modulation amplitude is increased. The growth of the modulation time leads to a statistically significant decrease in thymol oxidation currents. Thus, the best response of thymol has been achieved at a modulation amplitude of 100 mV and a modulation time of 25 ms.

**Figure 10:**
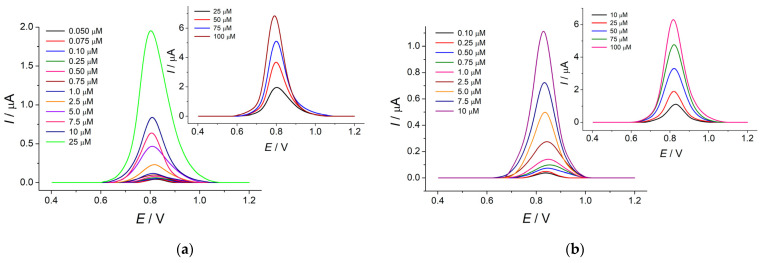
Baseline-corrected DPVs of isopropylmethylphenols at various concentrations at the polythymolphthalein/MWCNTs/GCE in Britton–Robinson buffer pH 2.0: (**a**) thymol; (**b**) carvacrol. Δ*E*_mod_ = 100 mV, *t*_mod_ = 25 ms, υ = 20 mV s^−1^.

Under these conditions, there are clear oxidation peaks of thymol and carvacrol at 0.81 and 0.83 V, respectively (Figure 10), the height of which are linearly dependent on the isopropylmethylphenols concentrations in the ranges of 0.050–25 and 25–100 µM for thymol and 0.10–10 and 10–100 µM for carvacrol with detection limits of 0.037 and 0.063 µM, respectively. The calibration graph parameters are presented in Table 2. The slopes of calibration plots indicate the high sensitivity of the isopropylmethylphenols response at the polythymolphthalein-modified electrode.

The analytical characteristics of thymol and carvacrol achieved are the best ones reported to date (Table 3).

The accuracy of the voltammetric method developed has been evaluated on the model solutions using the added–found method (Table 4). The relative standard deviation is less than 5% and confirms the absence of random errors in the determination. Recovery values of 99–100.1% testify to the high accuracy of thymol and carvacrol quantification. Furthermore, the relative standard deviation values indicate the perfect reproducibility of the results since the electrode surface renewal has been carried out after each measurement. The oxidation products are adsorbed at the electrode surface leading to its fouling and the significant decrease in isopropylmethylphenols oxidation currents at the second and following scans. Thus, only the single use of the electrode is possible.

The selectivity of the electrode response to isopropylmethylphenols has been tested using 1.0 µM thymol or carvacrol. Typical interferences such as inorganic ions (1000-fold excesses of K^+^, Mg^2+^, Ca^2+^, NO_3_^−^, Cl^−^, and SO_4_^2−^) and saccharides (100-fold excesses of glucose, sucrose, rhamnose, and arabinogalactan) are electrochemically inactive in the potential range studied and do not affect the oxidation peak of thymol or carvacrol. Ascorbic acid is oxidized at 0.52 V and 0.89 V at polythymolphthalein/MWCNTs/GCE, but the oxidation currents are low enough and the second peak is fully disappeared at 5.0 µM concentration. Therefore, there is no overlap between thymol or carvacrol and ascorbic acid oxidation peaks at an up to 5-fold excess of ascorbic acid (Figure S2a).

Since plants of the *Lamiaceae* family are the main sources of isopropylmethylphenols containing other phenolics, the potential interference effect of these compounds has been evaluated. Phenolic acids (gallic, caffeic, chlorogenic, and rosmarinic) are electroactive at polythymolphthalein/MWCNTs/GCE. Caffeic and chlorogenic acids are oxidized at 0.48 and 0.52 V, respectively, and their oxidation peaks do not overlap with the oxidation peak of thymol or carvacrol (the peak potential separation of 290–340 mV is achieved) at up to 50-fold excess of hydroxycinnamic acids in the mixture (Figure S2b,c). Rosmarinic acid gives a high oxidation peak at 0.46 V, but does not affect thymol or carvacrol determination (Figure S2d) at up to 10-fold excess. Gallic acid shows a two-step oxidation at 0.49 and 0.82 V (Figure S2e). The second oxidation step is fully overlapped with the thymol or carvacrol oxidation peak. However, the sensitivity of the gallic acid response is significantly lower than that of isopropylmethylphenols and its oxidation peaks have completely disappeared at 1.0 µM concentration. Therefore, gallic acid does not interfere with the determination of thymol or carvacrol up to equimolar ratio (Figure S2e), and a simple dilution of the sample can be applied to remove the interference effect of gallic acid. Quercetin is also oxidized at the polythymolphthalein/MWCNTs/GCE by two steps at 0.45 and 1.15 V. Nevertheless, no interference effect has been observed on thymol or carvacrol oxidation peaks at a 25-fold excess of quercetin (Figure S2f).

The data obtained confirm the high selectivity of the isopropylmethylphenols response at the polythymolphthalein/MWCNTs/GCE and the applicability of the electrode to plant material analysis.

### 3.6. Determination of Total Isopropylmethylphenols in Spices

The practical applicability of the voltammetric method developed has been demonstrated on thyme and oregano spices as the major source of isopropylmethylphenols [1]. Almost the same oxidation potentials of thymol and carvacrol make possible the determination of total isopropylmethylphenols as far as both of them are usually presented in plant materials.

**Figure 11:**
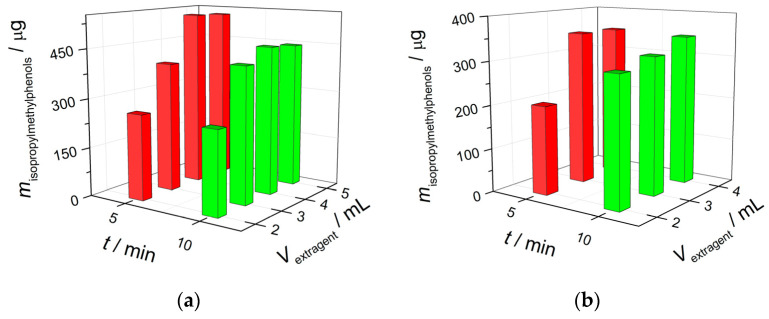
Isopropylmethylphenols extraction efficiency from spices: (**a**) oregano; (**b**) thyme. Data are based on the DPV for the extracts at the polythymolphthalein/MWCNTs/GCE in Britton–Robinson buffer pH 2.0.

Sonication-assisted extraction with methanol has been developed for the isopropylmethylphenols extraction. Variation of the extraction conditions (multiplicity, spice:extragent ratio, and extraction time) has shown that maximum efficiency is achieved by a single extraction for 5 min at 1:40 ratio for oregano and 1:30 for thyme (Figure 11). The results have been calculated using the thymol calibration graph as far as its response is more sensitive than that for carvacrol.

**Figure 12:**
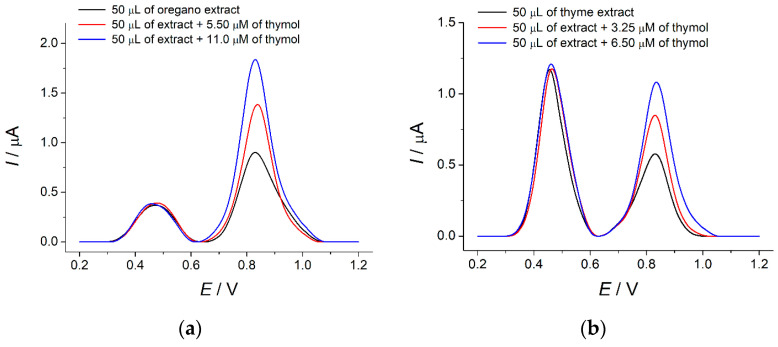
Baseline-corrected DPVs of spices extracts at the polythymolphthalein/MWCNTs/GCE in the Britton–Robinson buffer pH 2.0: (**a**) oregano extract with standard additions of thymol; (**b**) thyme extract with standard additions of thymol. Δ*E*_mod_ = 100 mV, *t*_mod_ = 25 ms, υ = 20 mV s^−1^.

Well-defined oxidation peaks at 0.43 and 0.83 V have been registered on the DPVs of oregano and thyme extracts (Figure 12). The second oxidation potential ideally coincides with that for thymol and carvacrol, which is also confirmed by the standard addition method (Figure 12). A proportional increase in oxidation currents at 0.83 V has been obtained after the addition of thymol. The recovery of 99–101% (Table S1) confirms the absence of matrix effects and the high accuracy of isopropylmethylphenols determination.

The determination of total isopropylmethylphenols in oregano and thyme spices is summarized in Table 5. Voltammetric data have been compared with those obtained by the independent spectrophotometric method [13]. A good agreement of the results obtained has been observed. A one-sample *t*-test indicates the absence of systematic errors of determination. The *F*-test confirms the similar accuracy of the both methods since the *F*-criterium values are less than the critical value.

The isopropylmethylphenol contents in oregano and spices are in agreement with those reported in the literature [50,68].

## 4. Conclusions

GCE modified with MWCNTs and electropolymerized thymolphthalein has been developed for the determination of total isopropylmethylphenols (thymol and carvacrol). The layer-by-layer combination of MWCNTs provides conductivity of the electrode, a high electroactive surface area, and significant improvement in the electron transfer rate. The structural similarity between polymeric coverage and isopropylmethylphenols and the porous structure of the electrode surface allow the obtaining of highly sensitive and selective responses of thymol and carvacrol at the polythymolphthalein-modified electrode. The analytical characteristics achieved are the best one among reported to date for both thymol and carvacrol. The electrode is simple in preparation, reliable, cost-effective, and can be applied in the analysis of spices. Screen-printed electrodes could be considered a possible platform for the fabrication of the electrodes applicable in routine practice.

## Figures and Tables

**Table 1:** Electroactive surface area and fitted electrochemical impedance parameters of the electrodes (*n* = 5; *p* = 0.95).

Electrode	*A* (mm^2^)	*R*_s_ (Ω) ^1^	*R*_et_ (kΩ) ^2^	*Q* (µΩ^−1^) ^3^	*n*	*W* (µΩ^−1^) ^4^	χ ^2,5^
GCE	8.9 ± 0.3	245 ± 5	72 ± 3	3.7 ± 0.2	0.789	–	0.028
MWCNTs/GCE	75 ± 2	199 ± 3	12.1 ± 0.9	3.6 ± 0.1	0.775	230 ± 5	0.015
Polythymolphthalein/MWCNTs/GCE	88 ± 5	112±5	7.3±0.1	0.704±0.09	0.889	225±10	0.021

^1^ Electrolyte resistance. ^2^ Electron transfer resistance. ^3^ Constant phase element. ^4^ Warburg impedance. ^5^ Fitting error.

**Table 2:** Calibration graph parameters of isopropylmethylphenols determination at the polythymolphthalein/MWCNTs/GCE in Britton–Robinson buffer pH 2.0.

Analyte	Linear Dynamic Range (µM)	*I* = *a* + *bc*		
		*a* ± SD (µA)	(*b* ± SD) × 10^−3^ (µA M^−1^)	*R* ^2^
Thymol	0.050–25	0.045 ± 0.001	80.3 ± 0.1	0.9999
	25–100	0.45 ± 0.07	64 ± 1	0.9992
Carvacrol	0.10–10	0.030 ± 0.002	94.8 ± 0.3	0.9999
	10–100	0.40 ± 0.04	59.6 ± 0.7	0.9994

**Table 3:** Figures of merit for the voltammetric determination of thymol and carvacrol at different modified electrodes.

Electrode	Method	Detection Limit (µM)	Linear Dynamic Range (µM)	Ref.
Thymol				
Nafion/MWCNTs/GCE	AdSDPV ^1^	0.05	0.5–5.0	[43]
Carboxylated MWCNTs-Sodium dodecyl sulfate/GCE	LSV ^2^	0.14	0.500–17.0; 17.0–150	[44]
Single-walled carbon nanotubes/Screen-printed electrode	DPV	4.0	5–90	[46]
Graphene oxide nanosheet/GCE	DPV	0.065	2–200	[47]
CeO_2_ nanoparticles-Graphene/GCE	DPV	0.050	0.10–18	[48]
CeO_2_ nanoparticles-Brij^®^ 35/GCE	DPV in Brij^®^ 35 medium	0.20	0.700–10.1; 10.1–606	[49]
Ag@C@Ag core–double shell spheres/GCE	AdDPV ^3^	0.0216	0.1–10	[51]
MnY nanozeolite/Carbon paste electrode	DPV	0.243	0.5–28	[52]
Polyacrylamide embedded graphite molecular imprinted polymer based electrode	DPV	0.043	0.5–10; 10–300	[56]
Polythymolphthalein/MWCNTs/GCE	DPV	0.037	0.050–25; 25–100	This work
Carvacrol				
MWCNTs–Sodium dodecyl sulfate/GCE	DPV	0.075	0.1–25; 25–150	[45]
La_2_O_3_/Co_3_O_4_ nanocomposite/Screen-printed electrode	DPV	1.0	10–800	[50]
Polythymolphthalein/MWCNTs/GCE	DPV	0.063	0.10–10; 10–100	This work

^1^ Adsorptive stripping DPV. ^2^ Linear sweep voltammetry. ^3^ Adsorptive DPV.

**Table 4:** Quantification of isopropylmethylphenols in model solutions using polythymolphthalein/MWCNTs/GCE in Britton–Robinson buffer pH 2.0. (*n* = 5; *p* = 0.95).

Isopropylmethylphenol	Added (µg)	Found (µg)	RSD (%)	Recovery (%)
Thymol	0.0376	0.0375 ± 0.0001	0.40	99.9 ± 0.5
	0.376	0.37 ± 0.01	2.5	99 ± 3
	1.88	1.86 ± 0.09	3.7	99 ± 5
	5.63	5.62 ± 0.05	0.72	99.7 ± 0.9
	18.8	18.8 ± 0.4	1.3	100 ± 2
	75.1	75.2 ± 0.7	0.62	100 ± 1
Carvacrol	0.0751	0.074 ± 0.004	0.47	99 ± 4
	0.563	0.562 ± 0.006	0.91	100 ± 1
	3.76	3.74 ± 0.06	1.3	99 ± 2
	7.51	7.52 ± 0.04	3.2	100.1 ± 0.5
	37.6	37.5 ± 0.6	0.62	100 ± 1
	75.1	75 ± 1	0.60	100 ± 1

**Table 5:** Quantification of the total content of isopropylmethylphenols in spices (*p* = 0.95).

Spice	Sample Number	Total Contents of Isopropylmethylphenols (mg g^−1^)				*t*-Test ^1^	*F*-Test ^2^
		Found by Voltammetry	RSD (%)	Found by Spectrophotometry	RSD (%)		
Oregano	1	5.4 ± 0.1	1.6	5.5 ± 0.5	3.8	1.24	6.09
	2	1.20 ± 0.02	0.62	1.21 ± 0.04	2.9	0.251	2.18
	3	6.5 ± 0.5	2.8	6.4 ± 0.9	5.4	0.619	3.57
Thyme	1	4.2 ± 0.1	1.0	4.3 ± 0.1	1.4	1.60	1.75
	2	2.9 ± 0.2	2.3	2.9 ± 0.3	4.0	0.996	3.01
	3	1.9 ± 0.1	2.7	2.0 ± 0.2	5.0	2.32	4.00

^1^ *t*_crit_ = 2.45 at *α* = 0.05 and *f* = 6. ^2^ *F*_crit_ = 6.59 at *α* = 0.05 and *f*_1_ = 4, *f*_2_ = 2.

## Data Availability

The data presented in this study are available in the electronic Supplementary Materials.

## Supplementary Materials

Supporting information can be downloaded at the following: https://www.mdpi.com/article/10.3390/mi14030636/s1, Figure S1: (a) Chronoamperometric curves of ferrocyanide ions in 0.1 M KCl at the bare GCE at 0.45 V; (b) Plot of *I* vs. *t* ^−½^ on the basis of chronoamperometric data. Figure S2: Effect of the ascorbic acid and natural phenolics on the voltammetric response of thymol at the polythymolphthalein/MWCNTs/GCE in Britton–Robinson buffer pH 2.0: (a) effect of 5.0 µM ascorbic acid; (b) effect of 50 µM caffeic acid; (c) effect of 50 µM chlorogenic acid; (d) effect of 10 µM rosmarinic acid; (e) effect of 10 and 1.0 µM gallic acid; (f) effect of 25 µM quercetin. Δ*E*_mod_ = 100 mV, *t*_mod_ = 25 ms, υ = 20 mV s^−1^; Table S1: Recovery of total isopropylmethylphenols in spices extracts at the polythymolphthalein/MWCNTs/GCE in Britton–Robinson buffer pH 2.0 (*n* = 5; *P* = 0.95).
