# Supplementary material for: Electrode Based on the MWCNTs and Electropolymerized Thymolphthalein for the Voltammetric Determination of Total Isopropylmethylphenols in Spices
Source: Micromachines (Basel). 2023 Mar 11;14(3):636. doi: 10.3390/mi14030636 (PMC10058491; doi:10.3390/mi14030636)
Supplement: Supplementary file 1 [file micromachines-14-00636-s001.zip › micromachines-2248599-supplementary.pdf]

Supplementary Materials

Electrode Based on the MWCNTs and Electropolymerized Thymolphthalein for the Voltammetric Determination of Total Isopropylmethylphenols in Spices

Natalia Chernousova and Guzel Ziyatdinova*

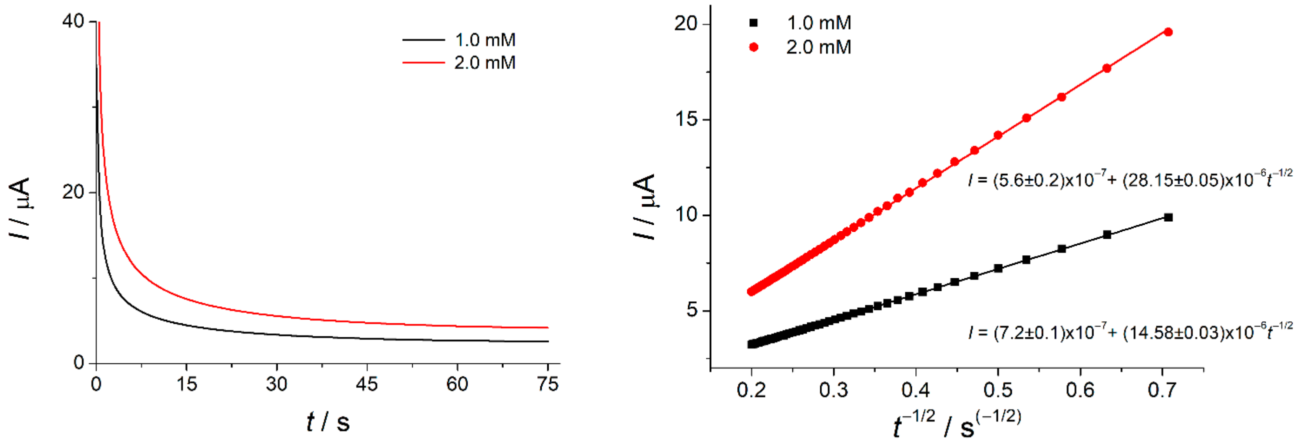

(**a**) (**b**)

Figure S1. (**a**) Chronoamperometric curves of ferrocyanide ions in 0.1 M KCl at the bare GCE at 0.45 V; (**b**) Plot of *I* vs. $t^{-½}$ on the basis of chronoamperometric data.

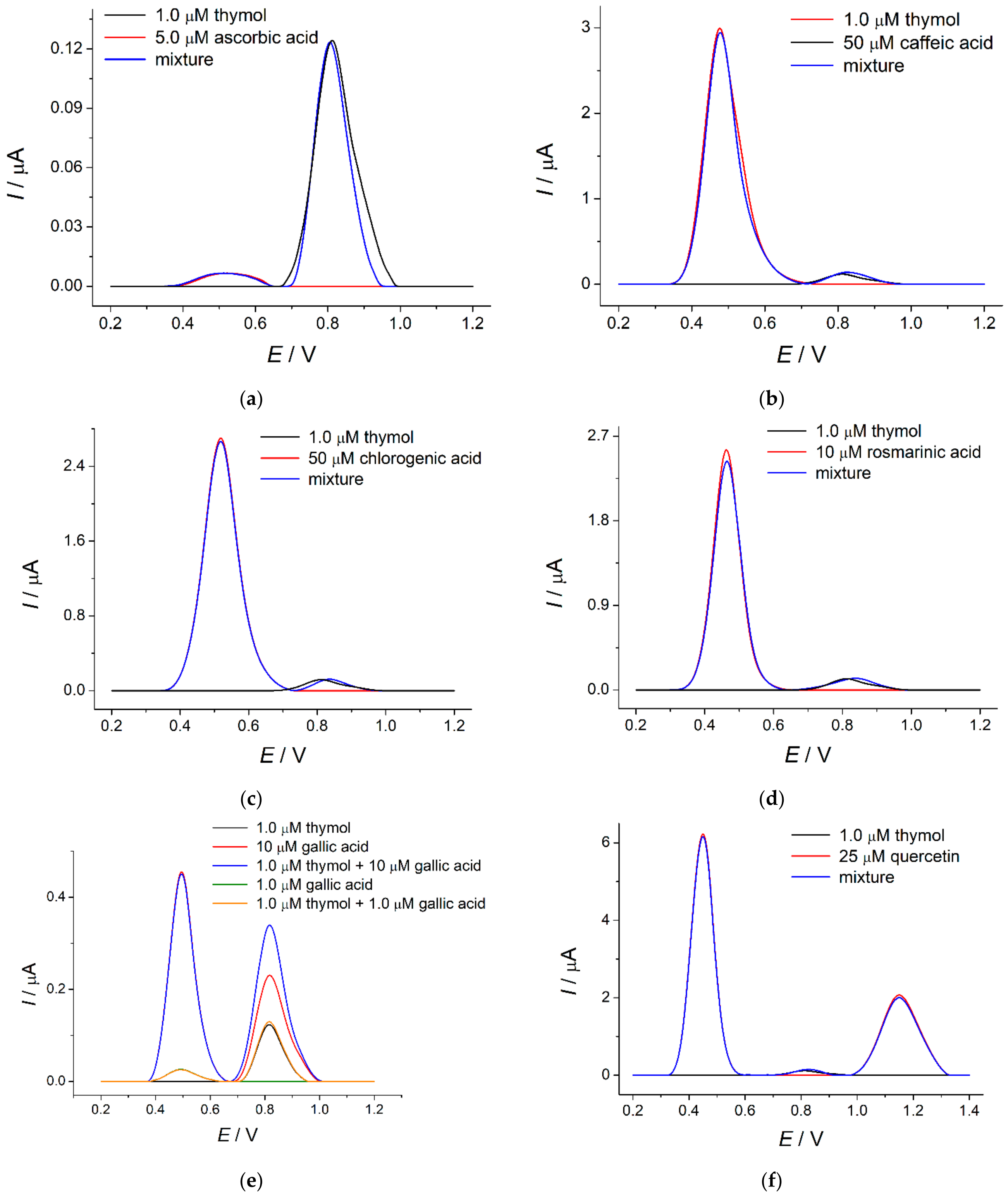

Figure S2. Effect of the ascorbic acid and natural phenolics on the voltammetric response of thymol at the polythymolphthalein/MWCNTs/GCE in Britton-Robinson buffer pH 2.0: (**a**) effect of 25 µM ascorbic acid; (**b**) effect of 50 µM caffeic acid; (**c**) effect of 50 µM chlorogenic acid; (**d**) effect of 10 µM rosmarinic acid; (**e**) effect of 10 and 1.0 µM gallic acid; (**f**) effect of 25 µM quercetin. ΔE_{mod} = 100 mV, t_{mod} = 25 ms, υ = 20 mV s^{-1}.

Table S1. Recovery of total isopropylmethylphenols in spices extracts at the polythymolphthalein/MWCNTs/GCE in Britton–Robinson buffer pH 2.0 (n = 5; P = 0.95).

Extract	Spiked (μM)	Found (μM)	RSD (%)	*R* (%)
Oregano	0	11 ± 2	3.0	
	5.50	16.6 ± 0.5	1.3	100 ± 2
	11.0	22.0 ± 0.2	3.4	99 ± 3
Thyme	0	6.5 ± 0.3	1.8	
	3.25	9.78 ± 0.03	0.17	100.5 ± 0.6
	6.50	13.1 ± 0.2	0.76	101 ± 2
